# Dietary Micronutrients Intake Status among Chinese Elderly People Living at Home: Data from CNNHS 2010–2012

**DOI:** 10.3390/nu11081787

**Published:** 2019-08-02

**Authors:** Zhen Liu, Liyun Zhao, Qingqing Man, Jingzhong Wang, Wenhua Zhao, Jian Zhang

**Affiliations:** National Institute for Nutrition and Health, Chinese Center for Disease Control and Prevention, 29 Nanwei Road, Xicheng District, Beijing 100050, China

**Keywords:** micronutrients, dietary, elderly, China

## Abstract

The aim of this study was to examine the status of usual dietary micronutrient intakes among Chinese elderly living at home. The data was based on China National Nutrition and Health Survey (CNNHS) 2010–2012. We analyzed data from the participants aged 60-year-old and more (*n* = 16,612) living at home, who provided dietary data on three days 24 h dietary survey combining with the household weighing method. The means and distribution percentiles for usual intakes of dietary micronutrients were estimated using the Multiple Source Method (MSM). The prevalence of inadequacy for the selected micronutrients was expressed using the proportion of individuals with usual intakes below the Estimated Average Requirement (EAR). For vitamin E, sodium and potassium, the means and the distribution of intakes were compared to the Adequate Intake (AI) level. Usual dietary intakes of most micronutrients were inadequate in the participants, especially folate, calcium, vitamin B_6_ and vitamin B_2_, with the prevalence of inadequacy more than 90%. However, dietary sodium intake was extremely high with an average usual intake of 4702 mg/day. The usual dietary intakes of all selected micronutrients in old males were higher than females, and the prevalence of inadequacy of most micronutrients was higher in old women (*p* < 0.01). The subjects aged 60–74 years tended to have higher usual dietary micronutrient intakes and lower prevalence of inadequate micronutrients than those aged 75 years and over (*p* < 0.01). Higher usual dietary intakes and lower prevalence of inadequacy of most micronutrients were found in the elderly living in the southern region (*p* < 0.01). The average usual intakes of most micronutrients declined with socioeconomic status. The prevalence of inadequate vitamin A, B_2_, C, calcium and selenium below EAR increased with socioeconomic status (*p* < 0.01, *p* for trend < 0.01). Thus, essential micronutrients insufficient intake is a public health concern among Chinese community-dwelling old population, especially the females, older people, the elderly in undeveloped areas or living in northern regions. Nutrition education and appropriate approach should be undertaken to address these problems.

## 1. Introduction

Micronutrients deficiency referred to as “hidden hunger” is a global health issue. It is supposed that over 2 billion people worldwide [1] are suffering from micronutrients deficiency and there are surprisingly high prevalence rates in developing countries and among vulnerable groups, in particular, among the elderly population [2]. 

Micronutrients deficiency contributes to the course from the normal aging process to the progression of chronic disease and impacts susceptibility and exacerbation of disease and disability of the elderly [2]. Many studies found the association between deficient micronutrients and detrimental health consequences as well as long-term impairment in the elderly, including decreased immunity [3], oxidative stress [4], anemia [5], neurodegenerative diseases [6,7], impaired cognitive performance [8], metabolic diseases [9], cancer [10], macular degeneration [11], hip fracture [12], cardiovascular diseases [13] and increased morbidity and mortality [14]. As a consequence, micronutrients deficiency is projected to cost around 0.8–2.5 per cent of the gross domestic product in some developing countries [15,16]. 

As its performance is not so obvious, micronutrients deficiency can only be diagnosed in the event of serious consequences. Thus, sufficient dietary micronutrients intake is the most essential, effective and economical approaches to primarily prevent micronutrients deficiency as well as the subsequent severe outcome. Accompanied with aging, physiologic changes and functional decline usually affect the ingestion, absorption and utilization of nutrients. Although considerable evidence was available regarding macronutrients intake and distribution [17,18], the basic data of dietary micronutrients intake in elderly population is limited. 

China has entered an aging society since 2000. Substantial demographic and socioeconomic changes in China have created significant health and nutrition challenges. It is very important to improve nutrition status of older people to promote their health and wellness. In some countries, several national studies and meta-analyses found that the older population is at risk of insufficient micronutrients intake [19,20,21], but there is a lack of research to estimate the micronutrients intake in Chinese elderly that might be suboptimal. In this study, based on the data from the China National Nutrition and Health Survey (CNNHS) 2010–2012, we evaluated the micronutrients nutritional status in Chinese elderly and compared them between/among different gender, ages, socioeconomic types and geographic regions. To our knowledge, this is the first time that micronutrients status and the potential inadequacies among Chinese home-living old adults are estimated based on results of the national representative survey.

## 2. Materials and Methods

### 2.1. Data Sources

**Figure 1:**
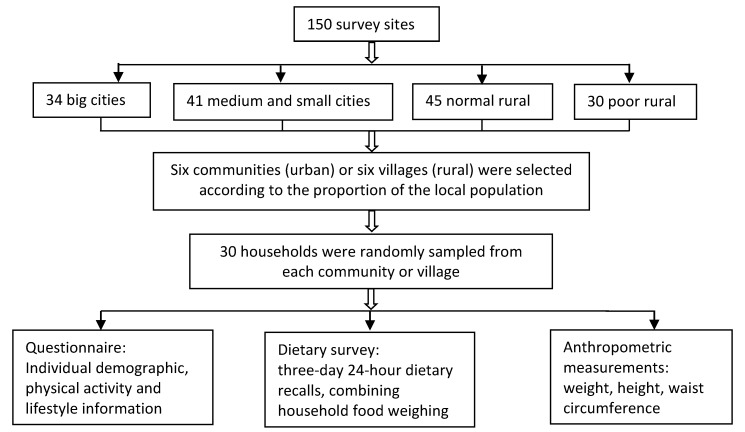
The flow diagram of the China National Nutrition and Health Survey (CNNHS) during 2010–2012.

In this study, we used data from the China National Nutrition and Health Survey (CNNHS) during 2010–2012 (Figure 1). The CNNHS was carried out on stratified multistage systematic clustered random sampling method with proportional to the population to form a representative sample of China as a whole, including 31 provinces, autonomous regions and municipalities. Random sampling method was conducted from six communities (urban) or six villages (rural) equal to the proportion of the local population at 150 survey sites from four different socioeconomic area types (big cities, medium and small-sized cities, normal rural and poor rural). Thirty households were randomly sampled from each community or village into the survey. The dwellers from these households were local residents or lived locally over 6 months. Adult participants that can walk independently to the survey site were included in the survey. The CNNHS was approved by the Ethical Committee of the National Institute for Nutrition and Food Safety of the Chinese Center for Disease Control and Prevention (2013(018)) [22]. All the participants provided written informed consent. We chose the subjects aged 60 years old and over into this study.

### 2.2. Subjects and Methods

In the CNNHS, questionnaires were designed to gather information on the individual socio-demographic characteristic, diet, physical activity, health and lifestyle status. Anthropometric measurements were taken to examine individual weight, height and waist circumference by trained staff. 

**Figure 2:**
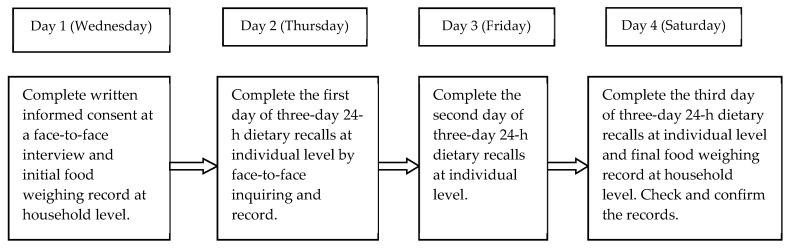
The representative flow of dietary survey in CNNHS 2010–2012.

The dietary survey was carried out by highly trained dietary staff inquiring and recording the information via a face-to-face interview, including three-day consecutive 24-hour dietary recalls at individual level, combining food weighing at household level (two weekdays and one weekend). The flow of representative dietary survey was shown in Figure 2. On each visit, all food and beverages consumed at home and away from home in the past 24 hour were described by the participants, including time of consumption, sources where the food was obtained, cooking method and portion size. Several visual aids, such as food models and pictures involving standard plates, bowls, cups, spoons, bottles and other common household utensils were provided to help the participants estimate the portion sizes consumed. If the older participants were unable to provide the information by themselves, the person who took care of them or prepared the dishes for them surrogated them to complete the diet survey. The amounts of edible oil and each ingredient (such as salt, sugar and other condiment) used at the household were measured with the use of a uniformly calibrated electronic dietary scale with a precision of 2 g. Meanwhile, the newly purchased and abandoned amounts of these cooking oil and condiments were asked to be recorded during the survey days. Then, the food names and amount during the dietary survey days were documented or calculated by the trained interviewers. Data regarding dietary nutrient intake did not include the consumption of nutrient supplements or medicines. Categorized food intake and dietary micronutrients intake were derived from both 24-hour recall data and household weighing data, and calculated by food codes and Chinese Food Composition Table [23,24]. Because of an absence of the data of vitamin B_12_ in the Chinese Food Composition Table, we used vitamin B_12_ content of the analogical food (including 1321 food items) of the United States Department of Agriculture (USDA) Food Composition Databases [25] and Standard Tables of Food Composition in Japan [26] to replace and estimate the values. 

A total of 17,042 participants aged 60 years and over had available data of food intake. The persons with energy intakes lower than 800 kcal (3346 kJ) per day per reference man (*n* = 183) or higher than 5000 kcal (20,910 kJ) per day per reference male (*n* = 224) and the persons who reported less than one day of dietary records (*n* = 23) were excluded. Finally, 16,612 subjects aged 60 years and over were involved in this study to be analyzed.

### 2.3. Statistical Analysis

All data were independently double entered by different people for subsequent validation using EpiData, version 3.1 (CDC, Atlanta, GA, USA). Any discrepancies were identified, checked against original records and corrected before data analysis. Data analysis was performed using SAS (Statistical Analysis System) for Windows V9.3 (SAS Institute, Cary, NC, USA).

The multiple source method (MSM) [27] was used to estimate the means and distribution percentiles for usual dietary micronutrient intake, which were proposed by the European Prospective Investigation Cancer and Nutrition (EPIC) and corrected for intra-individual variability of consumption by the shrinkage modeling. All participants were considered consumers of micronutrients. The subjects’ gender, age groups (60–74 years group and ≥75 years group), geographic region (north and south) and socioeconomic types (big cities, small and medium sized cities, normal rural and poor rural) were considered as covariates into the all separate modeling parts created for each micronutrient. Usual dietary micronutrients intakes were compared to age and sex-specific recommendations defined by Chinese Dietary Reference Intakes [28].

According to the definitions of DRIs (Dietary Reference Intakes) [29], the Estimated Average Requirement (EAR) as the daily intake value is estimated to meet the requirement in half of the apparently healthy individuals in a particular life stage or gender group. Recommended Dietary Allowance (RDA) is the average daily dietary intake level that is sufficient to meet the nutrient requirement of nearly all (97 to 98%) healthy individuals, i.e., EAR + 2 SD (Standard Deviation) of the EAR, when normally distributed. As for Adequate Intake (AI), it is the recommended intake level based on observed or experimentally determined approximations of nutrient intake by a group (or groups) of healthy people that are assumed to be adequate, used when an RDA cannot be determined. In this study, the prevalence of inadequate intake of micronutrients was estimated as the proportion of the elderly who had a micronutrient intake below the EAR, applied by the EAR cut point method, as recommended by the Institute of Medicine (IOM) [30]. Micronutrients were considered to be potential inadequacy if the prevalence of inadequate intakes was equal to or above 30% of the population for each sex or age [21]. For vitamin E, sodium and potassium, the usual intake was compared with the AIs in order to make a qualitative comparison since the EARs of these nutrients for the older age group were not established in China yet. If the intake was above the AI, a low prevalence of inadequacy was assumed. If the intake was below the AI, the inadequacy could not be determined [30]. Comparisons in terms of the prevalence of inadequate intake for each micronutrient between/among different groups was examined by using Chi-square test and the trend among four socioeconomic types was tested with Cochran-Armitage trend test. A two tailed *p* value < 0.05 was considered to be statistically significant. 

## 3. Results

### 3.1. Characteristic and Usual Dietary Energy Intake of the Subjects

A total of 16,612 participants aged 60-years-old and above were involved in this study. The average age was 68.81 with 81.4% of 60–74 years and 18.6% of 75 years and over. The proportion of males was 49.0% and 41.0% of the subjects were from the northern region. The usual dietary energy intake of the subjects was 1783 ± 617 kcal/day. Table 1 provides an overview of the demographic and socioeconomic characteristics and usual dietary energy intake of the participants.

### 3.2. Assessment of Dietary Micronutrients Intakes

The means, selected percentiles of usual dietary micronutrient intakes and the proportion of the subjects with inadequate intake below EAR for each micronutrient among Chinese elderly were shown in Table 2. The EARs or AIs for the selected vitamins and minerals for Chinese adults aged 60 years and over were shown in Appendix A Table A1 and Table A2 [28].

Dietary daily intakes of folate, calcium and vitamin B_6_ were found extremely low with the medians of 59.2 µgDFE/day, 301.4 mg/day and 0.4 mg/day, respectively, not reaching a quarter or a half of their EARs in the elderly. Moreover, the median usual dietary intake of most micronutrients was less than the EARs for each respective sex and age group, such as vitamin A, vitamin B_1_, vitamin B_2_, vitamin B_12_, vitamin C, magnesium, zinc (only for male) and selenium. For magnesium, the medians of usual dietary intake were lower than its specific EARs for respective age group, with 247.4 mg/day in 60–64 years group, 231.5 mg/day in 65–79 years group and 194.3 mg/day in 80 years and over group. The dietary daily intakes of zinc for female and iron was higher than the EARs for respective sex-age group. For example, the median of usual dietary zinc intake for old females was 7.7 mg/day, which was higher than the EAR of 6.1 mg/day. The median usual intake of vitamin E with 23.3 mgα-TE/day was more than the AI of vitamin E for the elderly of 14 mgα-TE/day which implied a low likelihood of vitamin E inadequacy for the subjects. The median of dietary daily intake of potassium (1017 mg/day) was less than the AI for the elderly (2000 mg/day). On the contrary, the median usual sodium intake was extremely high, much more than the AIs of sodium for each respective age group. The median of usual sodium intake in 60–80 years group, 3915 mg/day, was 1.8 folds higher than the AI of 1400 mg/day and that in the over 80 years group (3451 mg/day) was also high compared to the AI of 1300 mg/day. 

The prevalence of inadequacy of vitamin B_2_ for both sex, vitamin B_6_, folate and calcium was very high with the proportion of the elderly below the EARs more than 90% (vitamin B_2_: 95.5% for men, 92.6% for women; vitamin B_6_: 95.1%; folate: 99.4%; and calcium: 98.2%, respectively). Furthermore, potential inadequacies were also found in vitamin A, vitamin B_1_, vitamin B_12_, vitamin C, magnesium, zinc (only for men) and selenium since the percentages of inadequate intakes of the preceding micronutrients was over 30% (67.6%–89.3%) for each respective sex or age group. For example, although the EARs reduced with age, the proportion of the subjects with dietary magnesium intake below EAR still increased among 60–64 years group, 65–79 years group and 80 years and over years group, with 65.4%, 67.7% and 76.9%, respectively. On the other hand, the proportion of the elderly with inadequate dietary iron was 3.3% and the prevalence of inadequate zinc for women was 24.8%. Thus, potential inadequacies of iron in the elderly and zinc in the older women were not considered to exist. The percentage of the elderly with usual dietary sodium intake more than the WHO (World Health Organization) recommendation (2000 mg/day) reached 88.6% [31].

#### 3.2.1. Assessment of Dietary Micronutrients Intakes between the Two Genders

Table 2 also summarized the usual dietary micronutrient intakes and the percentage of the elderly with inadequate micronutrients between genders. The usual dietary intakes of all micronutrients were found to be slightly higher in older men than women. However, given the higher dietary micronutrient requirements in males, inadequate intakes of vitamin A, vitamin B_1_, vitamin B_2_ and zinc below the EARs were more common in elderly men than in elderly women (*p* < 0.001). In addition, the percentages of older women with inadequate vitamin B_6_, vitamin B_12_, vitamin C, calcium, magnesium, iron and selenium intakes were found higher than men (*p* < 0.001). There was no significant difference in the prevalence of inadequate folate between old men and women (*p* > 0.05). The percentage of old males with dietary sodium intake more than 2000 mg/day was higher than females (91.4% versus. 85.8%, *p* < 0.001).

#### 3.2.2. Assessment of Dietary Micronutrients Intakes between Different Age Groups

Usual intakes of all micronutrients were found higher in 60–74 years group compared to the 75 years and over group (Table 3). The proportion of individuals with inadequate intakes was estimated by comparison with EAR values for each respective age group (60–64 years, 65–79 years and ≥80 years) and gender. Although the micronutrients requirements were relatively low in the older people aged over 65-year-old or 80-year-old, the prevalence of inadequacy of most micronutrients was still much higher in the older people (≥75 years) (*p* < 0.001), except for vitamin B_12_, folate and calcium, with no significant difference between the two age groups (Table 3). The percentage of the elderly with usual dietary sodium intake more than 2000 mg/day was higher in the 60–74 years group than the 75 years and over group (89.2% versus 85.5%, *p* < 0.001).

#### 3.2.3. Assessment of Dietary Micronutrients Intakes between Northern and Southern Regions

Means, selected percentiles of usual dietary micronutrient intake and the percentage of micronutrients inadequacies in northern and southern region were shown in Table 4. Median usual dietary intakes of vitamin A, vitamin B_2_, vitamin B_6_, vitamin B_12_, vitamin C, folate, calcium and zinc were higher and the prevalence of inadequacies of these micronutrients was lower in the southern region (*p* < 0.05). However, the median usual dietary intakes of vitamin E, potassium, sodium, magnesium and selenium were much higher in the elderly living in the northern region. Meanwhile, the percentage of the participants with inadequate magnesium and selenium intakes was higher in the southern region (*p* < 0.05). In addition, there was no difference in the prevalence of inadequate vitamin B_1_ and iron between the two geographic regions (*p* > 0.05). With regards to the dietary sodium intake, the percentage of the subjects with usual sodium intake more than 2000 mg/day was higher in the north with 90.1% than the south with 87.5% (*p* < 0.001).

#### 3.2.4. Assessment of Dietary Micronutrients Intakes between in Different Socioeconomic Types

Usual intake of dietary micronutrients including vitamin A, vitamin B_2_, vitamin B_12_, vitamin C, vitamin E, calcium, potassium and selenium was found to be reduced among the socioeconomic area types. Additionally, the proportion of the subjects with inadequate intake of the preceding micronutrients exclusive of vitamin E and potassium increased with the socioeconomic (*p* < 0.01, *p* for trend < 0.01). On opposite terms, the usual dietary vitamin B_6_ intake increased with the four socioeconomic types and the prevalence of inadequate vitamin B_6_ declined accordingly (*p* < 0.01, *p* for trend < 0.01). The median usual dietary intake of vitamin B_1_, folate, magnesium and zinc was lowest in the subjects living in small and medium-sized cities, and the prevalence of inadequacy of vitamin B_1_, magnesium and zinc intake was highest in small and medium-sized cities (*p* < 0.001). As to folate, the prevalence of inadequate folate was lowest in normal rural (*p* < 0.001). There was no significant difference in the percentages of the elderly with inadequate iron intake among the four socioeconomic area types (*p* > 0.05). The median of usual dietary sodium intake and the percentage of the subjects with excess dietary sodium intake (≥2000 mg/day) were both highest in normal rural (4131 mg/day, 90.1%, *p* < 0.001). The results were shown in Table 5. 

## 4. Discussion

As far as we know, this is the first study to estimate the detailed status of usual dietary micronutrients intake and assess the potential inadequacies of micronutrients in Chinese elderly on national level. Moreover, this study also discovered the differences among geographic location and socioeconomic status except for sex and age. Until now, only one study calculated the usual nutrient intake and the prevalence of inadequate intake in adults based on the dataset from CNNHS in 2002 [32]. It highlighted the inadequate intake of calcium, zinc, selenium, magnesium, thiamine (vitamin B_1_) and riboflavin (vitamin B_2_), but the nutrient intake status among different age groups, living regions and socioeconomic types was not estimated [32]. In accordance with the results of the preceding study, overall findings of this present study indicated that the majority of Chinese old adults had potential inadequate intake of essential micronutrients, such as folate, calcium, vitamin A, vitamin B_6_ and vitamin B_2_, etc.

In this study, we selected 15 micronutrients including vitamins and minerals, which were essential for elderly health. Among the vitamin nutrients, the prevalence of inadequacy for folate was the highest in the elderly, and the usual dietary folate intake was extremely low, less than 60 μgDFE/day. Up to now, there has been no literature concerning to dietary folate intake and the prevalence of inadequacy in China. However, there was a report that a high dietary folate consumption of 797–847 μgDEF/day and a low prevalence of inadequate total dietary folate intake of 6.1–7.7% was found in the adults aged >50 years in the United States [33]. Similarly, results from a study in Brazil also pointed to a high average dietary folate intake (376.6 μgDEF/day) and a low prevalence of folate inadequacy (9.5%) among the subjects aged 60 years and over [34]. Though it was important to note that these countries have adopted national folic acid fortification policies, in other countries which have not launched these nutritional policies, the dietary folate intake among the elderly was also far higher than that in China. For example, in Poland, the folate intake among adults aged 60 and above was on the level of 133–284 μgDEF/day [35] and dietary folate with 356 μgDEF/day for men and 269 μgDEF/day for women in Irish old adults [36].

Similar to folate, usual dietary vitamin B_6_, B_1_, B_2_ and B_12_ intake was also very low among Chinese old adults, and the prevalence of inadequacy was more than 80%. Folate and vitamins B_2_, B_6_ and B_12_ participated in DNA repair, methylation and chromosome maintenance in one-carbon metabolism pathways [37]. Deficiency in folate, vitamins B_6_ and B_12_ resulted in homocysteine accumulation, consequently associated with hypertension, cardiovascular disease and cognition impairment. Globally, there were 13.7 million new stroke cases in 2016 and 40% of them occurred in China, about 5.51 million cases [38]. One report suggested that stroke remained the primary cause of cripples and death among Chinese adults [39]. The poor nutritional status of B vitamins might contribute to the high incidence of stroke in China. Inadequate vitamin B complex intake might be associated with low consumption of whole grains and cereals, legumes, vegetable, fruits and nuts, as well as certain animal food such as dairy and fish in China [40,41,42].

In the past 20 years, the consumption of cooking oil, meat and sugar increased rapidly while the coarse food, vegetables and fruits consumption decreased continuously in Chinese residents [41,43]. Thus, we found insufficient dietary vitamin C intake and adequate dietary vitamin E in Chinese elderly in this study which was quite different from those of the elderly in European countries [20]. From these results, inadequate vitamin A intake might be an important public health concern among Chinese home-living elderly people with the inadequacy prevalence of 78.9% for men and 74.6% for women. However, the majority of European countries had mean vitamin A intakes over 1000 μgRE/day for both men and women aged over 60 years and the proportion of intakes below the EAR was very low; less than 20% [20]. In the USA, the prevalence of inadequate intake for vitamin A was also less a concern since the percentage of individuals with vitamin A intakes below the EAR ranged from 9.8%–25.7% among adults aged over 50 years [19].

The prevalence of calcium inadequacy was the highest among the mineral nutrients in this study (98.2%) with the average usual dietary calcium intake of merely 338.3 mg/day in Chinese elderly. Data from NHANES 2007–2010 showed that calcium intake ranged from 814 mg/day for ages 51–70 years to 933 mg/day for ages ≥71 years and the prevalence of calcium inadequacy was 9.2%–51.4% for the respective age and sex groups [19]. Dairy products were one of the most important sources of calcium, but the consumption of dairy products was considerably low in Chinese elderly [41,44]. Selenium, magnesium and zinc (only for men) intake was also considered as potential inadequacy because of the prevalence of inadequacies more than 30% for each age-gender group. In comparison to the elderly population in most of European countries, the prevalence of calcium, magnesium, zinc and selenium inadequacies was much higher in Chinese elderly [20]. The percentage of inadequate iron was only 3.3% in Chinese old people, even lower than that in some European countries [20]. We considered that the planted-based dietary pattern and the increasing meat consumption (from 58.9 g/day in 1992, 78.6 g/day in 2002 to 89.7 g/day in 2012) were the main reasons for sufficient iron intake in China [41]. On the other side, it was important to note that there were 0.68% of the subjects in this study having excess iron intake over UL (Tolerable Upper Intake Level) of iron (42 mg/day) for the elderly in China. Moreover, one study reported that the prevalence of high iron store (serum ferritin > 200) was 50.6% among Chinese adults aged 60 years and over in large city [45]. There might exist a potential iron overconsumption among Chinese elderly. High iron intake, especially heme iron intake, and high iron store had been shown to be associated with diabetes [46,47,48], cancer [49] and cognitive impairment [50] in the Chinese population. Although dietary total iron intake among Chinese people had slightly decreased during the recent decade (from 23.3 mg/day in 2002 to 21.5 mg/day in 2012) [51], increasing dietary heme iron intake (existing only in meat and meat products) and the potential risk of iron overload, which still existed, became public health concerns. The average usual dietary potassium intake was about 1400 mg/day, far below the AI. Although the salt consumption reduced during recent years [41], the sodium intake of Chinese elderly was also far above the recommendation. In American adults aged over 70 years, the mean intakes of potassium and sodium were 2457 mg/day and 2717 mg/day, respectively [19]. Several studies suggested that high sodium intake and the sodium-potassium intake ratio was an independent predictor of stroke risk as well as a significant risk factor for cardiovascular disease and all-cause mortality [52,53,54,55]. Based on the recommendation of the WHO with sodium intake less than 2000 mg per day and potassium intake at least 3510 mg per day, the desirable dietary sodium-potassium ratio was approximately 1.0 (mmoL/mmoL) or lower [56]. Although a recent study demonstrated that combined moderate sodium intake (3–5 g/day) with a high potassium intake (2.1 g/day) and with a sodium-potassium-ratio less than 2.4–4.0 was associated with the lowest risk of mortality and cardiovascular events [57], the dietary sodium-potassium ratio of 5.7 in Chinese elderly was still significantly higher than the upper limit.

In this study, the findings showed that the usual dietary intakes of overall micronutrients analyzed among the women aged ≥60 years were less than the men, and the prevalence of inadequacies for the majority of micronutrients were higher in old female. The results were similar with those of the European study, as women and especially elderly women appeared to be the most vulnerable groups with additional risk of micronutrients deficiency [58]. Some studies addressed that under-nutrition were more common among women than men and suggested that the decline in energy and nutrients intakes appeared to be related to the women consuming smaller portions of food [2,59]. Due to the interference from oral health problems, impaired chewing and swallowing function as well as physiologic challenges associated with chronic and acute diseases and medication use [60], a well-balanced diet became a challenge for the older persons and the greater risk for micronutrients deficiency was found in adults aged ≥75 years.

Environmental, social and financial barriers faced by older adults might also interfere with adequate dietary micronutrient intake. In this study, for the majority of micronutrients, higher usual dietary intake and lower prevalence of inadequacies were found in the elderly living in the southern areas or cities. There were some dietary pattern differences between the southern and northern regions in China due to the geographical location, climate condition, food supply and dietary habits. In general, the southern dietary pattern was deemed a balanced dietary pattern with a high intake of vegetables, fruits, eggs, fish and meat [61,62]. Nevertheless, the northern diet in China was simple and had less diversity, mainly including wheat noodles, dumplings, steamed buns and flat cakes [63]. Dietary diversity predicted the adequacy of micronutrient intake. In addition, for most micronutrients analyzed in this study, a tendency of decreased usual dietary intakes was observed with a decline in socioeconomic levels, and the prevalence of inadequacies increased as the decreasing of socioeconomic situation accordingly. Many studies suggested that micronutrients were likely to be deficient in low- and middle-income countries or areas [64,65] and the income levels had an important influence on micronutrient intake [66,67]. Generally, people’s food pattern depended on several variables, such as income and cost of food, as well as awareness and knowledge about the nutritional value of each food. Therefore, the population with low education and income might be apt to have the risk of nutrients deficiency.

## 5. Limitations

There were several limitations in this study. Firstly, in CNNHS 2010–2012, the dietary survey only collected whether the subjects used dietary supplement but not investigated the specific details on the type, frequency and amount of supplement consumption as well as nutrient content of each supplement. Based on the prior analysis, only 2.1% of the subjects in this study had used a certain dietary supplement which was far lower compared to the rates of supplements use reported by the elderly in other countries with 41.0%–72.6% [68,69]. Thus, it was possible that this study might slightly underestimate micronutrient intakes in Chinese elderly; we thought the effect of supplement use was small among this population. Secondly, since there is lack of the vitamin B_12_ data in Chinese Food Composition Table at present, we used vitamin B_12_ content of the analogical food deriving from USDA Food Composition Databases [25] and Standard tables of Food Composition in Japan [26] to replace and estimate. This might be inaccurate for the estimation of vitamin B_12_ intake but we thought this rudiment of dietary vitamin B_12_ status for the first time was necessary to be reported due to the intimate relationship between vitamin B_12_ and folate [70]. Thirdly, usual dietary vitamin D intake could not be estimated in this study, since there is a lack of vitamin D data in the Chinese Food Composition Table. Furthermore, it was difficult to replace vitamin D content of food based on the food composition databases from other countries due to the vast difference of food (containing vitamin D) among Chinese people, especially commercial food and fortified food. In general, the main source of vitamin D is the synthesis mediated by UVB radiation and dietary vitamin D intake may not be a good proxy for vitamin D status [71,72]. Thus, we were more inclined to use serum 25-hydroxyvitamin D to assess vitamin D status. Finally, we have not detected a biomarker of these micronutrients in serum to estimate the prevalence of deficiency risk in this study. However, some studies suggested that individuals consuming an adequate diet based on the Estimated Average Requirement might have a lower risk of any deficiency than those with an inadequate diet [58]. Moreover, a positive aspect of this study was the use of MSM to estimate the usual intake of dietary micronutrients which was defined as the long-run average daily intake of a dietary component. MSM used a mix-effect modeling parts reducing the biases and effects of the variations of individual intake, providing better results regarding the knowledge of the participants’ dietary intake. In the future study, we would measure the biomarkers for micronutrients and contrast to the usual dietary micronutrients intake in this study.

## 6. Conclusions

In conclusion, Chinese elderly living at home have a suboptimal micronutrients intake, except for sodium, iron, zinc (only for women) and vitamin E. Women aged 60 years and over, older adults and the elderly living in the northern region or in poor rural areas are more vulnerable to micronutrients deficiency. However, there exists extremely excessive dietary sodium intake among Chinese elderly, in particular older males, younger elderly and those living in the northern China or in rural areas. Hence, there is an urgent need for nutrition education, nutritional policies and appropriate nationwide intervention programs for these groups of people to improve their diet quality to meet the micronutrients intake recommendations.

## Figures and Tables

**Table 1:** Characteristics and usual dietary energy intake of subjects in the study.

	Male	Female	Total
Age, y	68.92 ± 6.56	68.69 ± 6.86	68.81 ± 6.71
Socioeconomic types *, *n* (%)			
Big cities	2153 (26.4%)	2436 (28.8%)	4589 (27.6%)
Small and medium sized cities	2255 (27.7%)	2264 (26.8%)	4519 (27.2%)
Normal rural	2413 (29.6%)	2401 (28.4%)	4814 (29.0%)
Poor rural	1327 (16.3%)	1363 (16.0%)	2690 (16.2%)
Age group, *n* (%)			
60–74	6613 (81.2%)	6916 (81.7%)	13,529 (81.4%)
≥75	1535 (18.8)	1548 (18.3%)	3083 (18.6%)
Geographic Region **, *n* (%)			
North	3296 (40.5%)	3515 (41.5%)	6811 (41.0%)
South	4852 (59.5%)	4949 (58.5%)	9801 (59.0%)
Energy intake, Mean ± SD (kcal/day)			
60–64 year	2078 ± 678	1727 ± 534	1893 ± 631
65–79 year	1899 ± 605	1611 ± 561	1757 ± 603
≥80 year	1663 ± 546	1396 ± 535	1516 ± 557

*: Socioeconomic types: Big cities: the central urban areas of big cities including municipalities, six designated cities and capital cities (population more than one million). Small and medium-sized cities: downtown areas that excluded the big cities. Poor rural: the key poor counties of the national poverty alleviation plan identified by China. Normal rural: all the rest of the counties outside the poor rural areas. **: Geographic Region: Northern and Southern regions were divided by Qinling Mountain and Huaihe River.

**Table 2:** Usual dietary intake and the prevalence of inadequacy of micronutrients in the subjects aged 60 years and over between genders.

	Male			Female			Total		
	Mean ± SD	Median (P_25_, P_75_)	Below EAR (%)	Mean ± SD	Median (P_25_, P_75_)	Below EAR (%)	Mean ± SD	Median (P_25_, P_75_)	Below EAR (%)
Vitamin A (μg/day) ^&^	403.5 ± 312.8	326.2 (195.8, 521.1)	78.9 ^#^	369.9 ± 299.8	294.5 (173.5, 477.8)	74.6	386.4 ± 307.1	309.5 (183.6, 498.7)	76.7
Vitamin B_1_ (mg/day)	0.8 ± 0.3	0.8 (0.6, 1.1)	89.3 ^#^	0.7 ± 0.3	0.7 (0.5, 0.9)	86.0	0.8 ± 0.3	0.7 (0.5, 0.9)	87.7
Vitamin B_2_ (mg/day)	0.7 ± 0.3	0.7 (0.5, 0.8)	95.5 ^#^	0.6 ± 0.3	0.6 (0.4, 0.7)	92.6	0.7 ± 0.3	0.6 (0.5, 0.8)	94.0
Vitamin B_6_ (mg/day)	0.6 ± 0.4	0.5 (0.3, 0.8)	93.9	0.5 ± 0.4	0.4 (0.2, 0.7)	96.3 ^#^	0.5 ± 0.4	0.4 (0.3, 0.7)	95.1
Vitamin B_12_ (μg/day)	1.5 ± 1.9	0.9 (0.5, 1.7)	80.3	1.3 ± 1.7	0.8 (0.4, 1.5)	83.3 ^#^	1.4 ± 1.8	0.9 (0.4, 1.6)	81.8
Vitamin C (mg/day)	73.4 ± 41.2	66.2 (44.4, 94.4)	68.8	67.4 ± 39.2	60.4 (39.7, 86.9)	73.1 ^#^	70.3 ± 40.3	63.1 (41.9, 90.6)	71.0
Folate (μg/day) ^&^	80.2 ± 65.4	63.7 (34.9, 108.4)	99.3	69.1 ± 57.1	55.8 (30.3, 92.3)	99.5	74.5 ± 62.4	59.2 (32.1, 100.5)	99.4
Vitamin E (mg/day) *^,&^	32.4 ± 35.8	25.4 (16.2, 38.2)	-	27.1 ± 34.4	21.5 (14.2, 32.1)	-	29.5 ± 35.2	23.3 (14.9, 35.4)	-
Calcium (mg/day)	356.7 ± 177.1	319.8 (235.2, 438.9)	97.8	320.1 ± 165.8	284.8 (207.4, 394.6)	98.5 ^#^	338.3 ± 173.3	301.4 (220.3, 417.4)	98.2
Potassium (mg/day) *	1490 ± 569	1406 (1097, 1771)	-	1312 ± 512	1234 (954, 1575)	-	1400 ± 548	1317 (1017, 1680)	-
Sodium (mg/day) *	5128 ± 4999	4255 (3009, 5931)	-	4292 ± 4432	3569 (2542, 4946)	-	4702 ± 4739	3876 (2741, 5473)	-
Magnesium (mg/day)	264.8 ± 95.1	251.2 (198.9, 313.1)	61.2	230.6 ± 83.5	217.4 (172.1, 275.7)	73.7 ^#^	247.4 ± 91.2	234.3 (184.7, 294.5)	67.5
Iron (mg/day)	19.4 ± 6.9	18.5 (14.9, 22.6)	2.0	16.8 ± 6.1	15.9 (12.7, 19.7)	4.6 ^#^	18.1 ± 6.6	17.1 (13.6, 21.2)	3.3
Zinc (mg/day)	9.5 ± 3.3	9.4 (7.2, 11.3)	67.6 ^#^	8.2 ± 3.0	7.7 (6.1, 9.7)	24.8	8.8 ± 3.2	8.3 (6.6, 10.5)	45.8
Selenium (mg/day)	40.4 ± 18.3	37.5 (28.1, 49.3)	76.6	34.8 ± 16.8	32.1 (23.7, 42.5)	85.5 ^#^	37.6 ± 17.8	34.6 (25.7, 46.1)	81.1

*: Compared to AI; ^#^ Chi-square test for categorized variables, *p* < 0.05. ^&^: μg retinol Retinol Activity Equivalents (RAE) per day; μg Dietary Folate Equivalent (DFE) per day; mg α-tocopherol equivalent (TE) per day.

**Table 3:** Usual dietary intake and the prevalence of inadequate micronutrients in the elderly between different age groups.

	60–74 years			≥75 years		
	Mean ± SD	Median (P_25_, P_75_)	Below EAR (%)	Mean ± SD	Median (P_25_, P_75_)	Below EAR (%)
Vitamin A (μg/day) ^&^	390.4 ± 307.8	313.9 (185.4, 505.9)	76.1	368.8 ± 301.3	296.2 (175.4, 462.5)	79.6 ^#^
Vitamin B_1_ (mg/day)	0.8 ± 0.3	0.7 (0.6, 0.9)	86.5	0.7 ± 0.3	0.6 (0.5, 0.8)	92.7 ^#^
Vitamin B_2_ (mg/day)	0.7 ± 0.3	0.6 (0.5, 0.8)	93.7	0.6 ± 0.3	0.6 (0.4, 0.7)	95.4 ^#^
Vitamin B_6_ (mg/day)	0.5 ± 0.4	0.5 (0.3, 0.8)	94.9	0.5 ± 0.4	0.4 (0.2, 0.7)	96.3 ^#^
Vitamin B_12_ (μg/day)	1.4 ± 1.8	0.9 (0.4, 1.6)	81.6	1.3 ± 1.7	0.8 (0.4, 1.5)	82.8
Vitamin C (mg/day)	72.1 ± 40.5	65.2 (43.5, 92.5)	69.5	62.4 ± 38.3	54.3 (35.7, 81.3)	77.5 ^#^
Folate (μg/day) ^&^	75.5 ± 62.5	60.6 (32.9, 101.9)	99.4	69.9 ± 57.1	56.9 (30.4, 93.4)	99.3
Vitamin E (mg/day) *^,&^	30.7 ± 38.1	24.1 (15.5, 36.1)	-	24.4 ± 17.5	20.3 (13.1, 30.2)	-
Calcium (mg/day)	342.4 ± 172.6	305.8 (225.1, 421.1)	98.3	318.6 ± 168.5	281.5 (200.8, 401.2)	97.9
Potassium (mg/day) *	1432 ± 549	1346 (1054, 1714)	-	1256 ± 518	1165 (897, 1523)	-
Sodium (mg/day) *	4784 ± 4833	3953 (2794, 5563)	-	4345 ± 4283	3595 (2512, 5076)	-
Magnesium (mg/day)	253.4 ± 90.7	239.9 (191.4, 300.3)	66.1	221.3 ± 87.4	206.6 (160.6, 267.5)	74.1 ^#^
Iron (mg/day)	18.5 ± 6.6	17.5 (14.1, 21.7)	2.5	16.1 ± 6.4	15.1 (11.8, 19.1)	6.9 ^#^
Zinc (mg/day)	9.3 ± 3.2	8.5 (6.8, 10.7)	43.3	7.9 ± 3.2	7.4 (5.6, 9.6)	57.0 ^#^
Selenium (mg/day)	38.4 ± 18.1	35.5 (26.4, 47.2)	80.1	33.8 ± 16.2	31.0 (22.1, 41.6)	85.7 ^#^

*: Compared to AI; ^#^ Chi-square test for categorized variables, *p* < 0.05. ^&^: μg retinol Retinol Activity Equivalents (RAE) per day; μg Dietary Folate Equivalent (DFE) per day; mg α-tocopherol equivalent (TE) per day.

**Table 4:** Usual dietary intake and the prevalence of inadequate micronutrients in the elderly between northern and southern regions.

	North			South		
	Mean ± SD	Median (P_25_, P_75_)	Below EAR (%)	Mean ± SD	Median (P_25_, P_75_)	Below EAR (%)
Vitamin A (μg/day) ^&^	308.2 ± 225.4	256.4 (152.7, 400.9)	84.2 ^#^	440.8 ± 345.2	357.6 (212.5, 568.9)	71.6
Vitamin B_1_ (mg/day)	0.8 ± 0.3	0.7 (0.5, 0.9)	87.5	0.8 ± 0.3	0.7 (0.5, 0.9)	87.7
Vitamin B_2_ (mg/day)	0.6 ± 0.3	0.6 (0.5, 0.8)	94.8 ^#^	0.7 ± 0.3	0.6 (0.5, 0.8)	93.5
Vitamin B_6_ (mg/day)	0.4 ± 0.3	0.3 (0.2, 0.5)	99.0 ^#^	0.6 ± 0.4	0.6 (0.3, 0.9)	92.4
Vitamin B_12_ (μg/day)	0.9 ± 1.0	0.6 (0.3, 1.1)	88.2 ^#^	1.7 ± 2.3	1.1 (0.5, 2.0)	77.4
Vitamin C (mg/day)	66.8 ± 39.3	59.3 (39.1, 85.8)	74.4 ^#^	72.8 ± 39.3	65.8 (44.1, 93.7)	68.7
Folate (μg/day) ^&^	54.5 ± 37.2	46.7 (29.3, 70.1)	99.9 ^#^	88.2 ± 71.7	75.3 (38.1, 117.9)	99.0
Vitamin E (mg/day) *^,&^	33.6 ± 46.4	25.5 (17.1, 38.5)	-	26.7 ± 24.1	21.7 (13.6, 32.7)	-
Calcium (mg/day)	324.3 ± 158.2	290.3 (215.6, 398.7)	98.6 ^#^	347.6 ± 181.5	309.4 (223.4, 430.7)	97.9
Potassium (mg/day) *	1404 ± 541	1324 (1025, 1680)	-	1396 ± 554	1312 (1014, 1679)	-
Sodium (mg/day) *	4897 ± 5092	4079 (2878, 5706)	-	4567 ± 4471	3757 (2667, 5293)	-
Magnesium (mg/day)	258.7 ± 96.5	244.3 (192.2, 308.2)	63.6	239.6 ± 86.2	226.6 (179.2, 285.4)	70.3 ^#^
Iron (mg/day)	18.3 ± 6.7	17.1 (13.6, 21.1)	3.4	18.1 ± 6.6	17.1 (13.7, 21.3)	3.3
Zinc (mg/day)	8.1 ± 2.8	7.7 (6.2, 9.4)	53.3 ^#^	9.3 ± 3.4	8.9 (6.9, 11.2)	40.6
Selenium (mg/day)	38.2 ± 15.5	36.4 (27.3, 46.9)	80.0	37.1 ± 19.3	33.7 (24.7, 45.4)	81.9 ^#^

*: Compared to AI; ^#^ Chi-square test for categorized variables, *p* < 0.05. ^&^: μg retinol Retinol Activity Equivalents (RAE) per day; μg Dietary Folate Equivalent (DFE) per day; mg α-tocopherol equivalent (TE) per day.

**Table 5:** Usual dietary intake and the prevalence of inadequacy of micronutrients in the subjects aged 60 years and over in different socioeconomic area types.

	Big Cities			Small and Medium-Sized Cities			Normal Rural			Poor Rural		
	Mean ± SD	Median (P_25_, P_75_)	Below EAR (%)	Mean ± SD	Median (P_25_, P_75_)	Below EAR (%)	Mean ± SD	Median (P_25_, P_75_)	Below EAR (%)	Mean ± SD	Median (P_25_, P_75_)	Below EAR (%)
Vitamin A (μg/day) ^&,$^	468.6 ± 294.3	401.2 (264.6, 592.8)	66.6 ^#^	430.1 ± 295.8	360.1 (228.6, 549.5)	72.0 ^#^	342.5 ± 300.8	273.4 (166.8, 425.8)	83.3 ^#^	246.3 ± 231.9	176.4 (100.9, 310.2)	90.3
Vitamin B_1_ (mg/day)	0.8 ± 0.3	0.7 (0.5, 0.9)	88.9	0.7 ± 0.3	0.7 (0.5, 0.8)	91.1 ^#^	0.8 ± 0.3	0.7 (0.6, 1.1)	85.5	0.8 ± 0.3	0.8 (0.6, 1.0)	83.6
Vitamin B_2_ (mg/day) ^$^	0.8 ± 0.3	0.7 (0.6, 0.9)	88.2 ^#^	0.7 ± 0.3	0.6 (0.5, 0.8)	94.5 ^#^	0.6 ± 0.2	0.6 (0.4, 0.7)	96.8 ^#^	0.5 ± 0.2	0.5 (0.4, 0.7)	98.4
Vitamin B_6_ (mg/day) ^$^	0.5 ± 0.3	0.4 (0.3, 0.6)	97.7 ^#^	0.5 ± 0.3	0.4 (0.2, 0.6)	97.6 ^#^	0.6 ± 0.4	0.6 (0.3, 0.9)	93.1 ^#^	0.6 ± 0.5	0.5 (0.2, 0.9)	90.1
Vitamin B_12_ (μg/day) ^$^	1.8 ± 1.7	1.3 (0.8, 2.3)	73.1 ^#^	1.6 ± 2.5	0.9 (0.5, 1.7)	80.9 ^#^	1.2 ± 1.6	0.7 (0.4, 1.4)	84.8 ^#^	0.6 ± 0.7	0.4 (0.2, 0.8)	93.1
Vitamin C (mg/day) ^$^	76.9 ± 42.8	69.4 (47.3, 98.9)	66.2 ^#^	72.4 ± 39.9	65.4 (43.6, 93.5)	69.1 ^#^	66.2 ± 38.7	58.8 (39.6, 83.7)	75.3	63.1 ± 37.1	56.1 (36.2, 83.6)	74.9
Folate (μg/day) ^&^	67.5 ± 48.8	57.3 (35.1, 87.3)	99.6	62.8 ± 53.7	48.6 (27.3, 82.8)	99.4	86.2 ± 73.2	75.3 (37.4, 113.9)	99.0 ^#^	84.7 ± 67.1	67.1 (32.3, 120.9)	99.7
Vitamin E (mg/day) *^,&^	27.5 ± 20.5	23.5 (16.2, 33.3)	-	28.3 ± 21.7	23.2 (15.4, 35.2)	-	28.1 ± 27.8	22.7 (14.2, 34.4)	-	37.6 ± 30.7	24.1 (13.2, 40.2)	-
Calcium (mg/day) ^$^	422.4 ± 193.7	393.1 (286.1, 516.1)	96.5 ^#^	353.7 ± 172.8	320.4 (239.8, 431.6)	97.9 ^#^	290.3 ± 129.3	265.6 (204.2, 346.7)	99.3 ^#^	250.7 ± 116.9	226.6 (169.5, 308.1)	99.7
Potassium (mg/day) *	1555 ± 596	1464 (1138, 1861)	-	1366 ± 530	1300 (990, 1653)	-	1351 ± 529	1261 (986, 1617)	-	1277 ± 466	1220 (954, 1523)	-
Sodium (mg/day) *	4287 ± 3489	3663 (2665, 5051)	-	4668 ± 4784	3859 (2756, 5428)	-	5063 ± 5739	4131 (2914, 5806)	-	4823 ± 4517	3858 (2648, 5708)	-
Magnesium (mg/day)	256.1 ± 95.3	241.5 (191.1, 303.1)	64.3	236.3 ± 86.6	223.3 (175.5, 282.9)	72.2 ^#^	244.1 ± 85.4	231.6 (184.4, 290.2)	68.2	257.4 ± 97.8	243.6 (189.5, 304.8)	64.1
Iron (mg/day)	19.1 ± 7.2	18.3 (14.3, 22.3)	2.9	17.7 ± 6.4	16.7 (13.4, 20.7)	3.4	17.6 ± 6.3	16.7 (13.3, 20.7)	3.5	18.2 ± 6.3	17.2 (13.6, 21.5)	3.6
Zinc (mg/day)	8.9 ± 3.1	8.5 (6.8, 10.5)	42.6	8.6 ± 3.1	8.2 (6.4, 10.2)	48.3 ^#^	8.8 ± 3.2	8.3 (6.6, 10.5)	46.0	8.9 ± 3.6	8.3 (6.2, 11.2)	46.9
Selenium (mg/day) ^$^	42.1 ± 17.7	39.2 (30.4, 50.8)	74.9 ^#^	36.9 ± 17.9	34.3 (25.2, 44.9)	82.8 ^#^	35.9 ± 18.8	33.2 (24.3, 44.3)	83.3 ^#^	33.9 ± 14.5	31.3 (23.5, 41.7)	85.2

*: Compared to AI; ^#^ Chi-square test for categorized variables, *p* < 0.05; ^$^: Cochran-Armitage trend test for categorized variables, *p* < 0.05. ^&^: μg retinol Retinol Activity Equivalents (RAE) per day; μg Dietary Folate Equivalent (DFE) per day; mg α-tocopherol equivalent (TE) per day.

## Appendix A

**Table A1:** EARs or AIs for selected vitamins for Chinese elderly *.

	Vitamin A ^&^	Vitamin B1	Vitamin B2	Vitamin B6	Vitamin B12	Folate ^&^	Vitamin C	Vitamin E ^&^
	EAR (μg/day)	EAR (mg/day)	EAR (mg/day)	EAR (mg/day)	EAR (μg/day)	EAR (μg/day)	EAR (μg/day)	AI (mg/day)
Men								
50–64	560	1.2	1.2	1.3	2.0	320	85	14
65–79	560	1.2	1.2	1.3	2.0	320	85	14
≥80	560	1.2	1.2	1.3	2.0	320	85	14
Female								
50–64	480	1.0	1.0	1.3	2.0	320	85	14
65–79	480	1.0	1.0	1.3	2.0	320	85	14
≥80	480	1.0	1.0	1.3	2.0	320	85	14

* Chinese Dietary Reference Intakes (DRIs) 2013. ^&^: μg retinol Retinol Activity Equivalents (RAE) per day; μg Dietary Folate Equivalent (DFE) per day; mg α-tocopherol equivalent (TE) per day. EARs: Estimated Average Requirements; AI: Adequate Intake.

**Table A2:** EARs or AIs for selected minerals for Chinese elderly *.

	Calcium	Potassium	Sodium	Magnesium	Iron	Zinc	Selenium
	EAR (mg/day)	AI (mg/day)	AI (mg/day)	EAR (mg/day)	EAR (mg/day)	EAR (mg/day)	EAR (μg/day)
Men							
50–64	800	2000	1400	280	9	10.4	50
65–79	800	2000	1400	270	9	10.4	50
≥80	800	2000	1300	260	9	10.4	50
Female							
50–64	800	2000	1400	280	9	6.1	50
65–79	800	2000	1400	270	9	6.1	50
≥80	800	2000	1300	260	9	6.1	50

* Chinese Dietary Reference Intakes (DRIs) 2013. EARs: Estimated Average Requirements; AI: Adequate Intake.
